# Immunogenicity and protective efficacy of a *Salmonella* Enteritidis *sptP* mutant as a live attenuated vaccine candidate

**DOI:** 10.1186/s12917-017-1115-3

**Published:** 2017-06-24

**Authors:** Zhijie Lin, Peipei Tang, Yang Jiao, Xilong Kang, Qiuchun Li, Xiulong Xu, Jun Sun, Zhiming Pan, Xinan Jiao

**Affiliations:** 1 Jiangsu Key Laboratory of Zoonosis, Jiangsu Co-Innovation Center for Prevention and Control of Important Animal Infectious Diseases and Zoonosis, MOA Key Laboratory of Prevention and Control of Biological Hazard Factors (Animal Origin) for Agrifood Safety and Quality, MOE Joint International Research Laboratory of Agriculture and Agri-product Safety, Yangzhou University, Yangzhou, 225001 China; 2 Division of Gastroenterology and Hepatology, College of Medicine, University of Illinois at Chicago, Chicago, 60612 USA; 3 Center for Comparative Medicine, Animal Infectious Disease Laboratory, College of Veterinary Medicine, Yangzhou University, Yangzhou, 225001 China; 4 Department of Anatomy and Cell Biology, Rush University Medical Center, Chicago, 60612 USA

**Keywords:** *Salmonella* Enteritidis, SptP, Vaccine, Immunogenicity, Immune protection

## Abstract

**Background:**

*Salmonella enterica* serovar Enteritidis (*S*. Enteritidis) is a highly adaptive pathogen in both humans and animals. As a *Salmonella* Type III secretion system (T3SS) effector, *Salmonella* protein tyrosine phosphatase (SptP) is critical for virulence in this genus. To investigate the feasibility of using C50336Δ*sptP* as a live attenuated oral vaccine in mice, we generated the *sptP* gene deletion mutant C50336*ΔsptP* in *S.* Enteritidis strain C50336 by λ-Red mediated recombination and evaluated the protective ability of the *S*. Enteritidis *sptP* mutant strain C50336Δ*sptP* against mice salmonellosis.

**Results:**

We found that C50336Δ*sptP* was a highly immunogenic, effective, and safe vaccine in mice. Compared to wild-type C50336, C50336Δ*sptP* showed reduced virulence as confirmed by the 50% lethal dose (LD_50_) in orally infected mice. C50336Δ*sptP* also showed decreased bacterial colonization both in vivo and in vitro. Immunization with C50336Δ*sptP* had no significant effect on body weight and did not result in obvious clinical symptoms relative to control animals treated with phosphate-buffered saline (PBS), but induced humoral and cellular immune responses at 12 and 26 days post inoculation. Immunization with 1 × 10^8^ colony-forming units (CFU) C50336Δ*sptP* per mouse provided 100% protection against subsequent challenge with the wild-type C50336 strain, and immunized mice showed mild and temporary clinical symptoms as compared to those of control group.

**Conclusions:**

These results demonstrate that C50336Δ*sptP* can be a live attenuated oral vaccine for salmonellosis.

## Background

*Salmonella spp*. is a Gram-negative, facultative anaerobe and intracellular pathogen in both humans and animals. Infection by *Salmonella* is a major public health problem [1], causing an estimated 93.8 million illnesses and 155,000 deaths each year worldwide [2] and more than 1 million illnesses and 350 deaths each year in the U.S. [3]. In the past 20 years, *Salmonella enterica* serovar Enteritidis (*S.* Enteritidis) has been one of the most common serotypes in salmonellosis in humans despite the implementation of control and prevention measures [4]. Humans can be infected with *Salmonella* via consumption of contaminated pork, beef, poultry, and eggs or contact with fecal matter in places with poor sanitation. Salmonellosis in humans is characterized by abdominal pain, diarrhea, nausea, vomiting, fever, and headache [5].

Salmonellosis treatment and protection strategies include antimicrobial therapy and vaccination, but emergence of multidrug-resistant strains is becoming a serious global problem [6, 7]. Vaccines based on inactivated bacteria can potentially prevent salmonellosis [8]; however, attenuated live vaccines generated by deletion of various identified *Salmonella* virulence genes have higher immunogenicity and greater efficacy than killed bacteria [9–14].

*Salmonella* protein tyrosine phosphatase (SptP) is a *Salmonella* T3SS effector protein encoded in *Salmonella* pathogenicity islands (SPI)-1. The SptP protein has an N-terminal domain that acts as a GTPase-activating protein for Cdc42 and Rac1, mediating alterations in the actin cytoskeleton of host cells [15], as well as a C-terminal domain that inhibits mitogen-activated protein kinase and extracellular signal-regulated kinase signaling [16]. SptP also suppresses interleukin-8 (IL-8) production and consequently the inflammatory response in hosts, thereby promoting *Salmonella* invasion and intracellular replication [17, 18]. In a mouse infection model, SptP suppressed the degranulation of mast cells and blocked neutrophil recruitment [19].

In this study, we generated the *sptP* gene deletion mutant C50336Δ*sptP* in *S.* Enteritidis strain C50336 by λ-Red mediated recombination [20]. The growth characteristics of C50336Δ*sptP* were similar to those of wild-type C50336. We therefore investigated the feasibility of using C50336Δ*sptP* as a live attenuated oral vaccine in mice by evaluating virulence, changes in body weight and clinical symptoms, bacterial persistence, immune responses, and protective efficacy.

## Methods

### Bacterial strains and cells lines

The wild-type *S.* Enteritidis strain C50336 was obtained from the National Institute for the Control of Pharmaceutical and Biological Products (Beijing, China). The *sptP* deletion mutant strain C50336Δ*sptP* was constructed by λ-Red-mediated recombination as previously described [20]. Briefly, the sequence of the chloramphenicol resistance cassette (Cm^R^) was amplified from plasmid pKD3, including 39-bp homology extensions at the 5′ and 3′ ends of the *sptP* gene (primers: forward 5′-tgaatcagcaggaagtgctcaaaaacatactgcaggaatgtgtaggctggagctgcttc −3′; reverse 5′-cttactttcagatagttctaaaagtaagctatgtttttaatgggaattagccatggtcc −3′). PCR products were purified and transferred into C50336 cells containing plasmid pKD46 by electroporation. Recombinant C50336-Cm^R^ cells grown on Luria-Bertani (LB) agar plates were selected for both Cm^R^ and ampicillin resistance (Amp^R^). Allelic replacement of *sptP* with the Cm cassette was verified by PCR analysis (primers: forward 5′-atccgaactactttacgc-3′; reverse 5′-tgaatggtattctactgg-3′) and DNA sequencing. The cassette was then excised by introducing the Flp recombinase-expressing vector pCP20. Biochemical tests were performed using the API 20E identification kit (BioMérieux, Lyon, France) and VITEK 2 Gram-negative bacilli test (BioMérieux) according to the manufacturer’s protocol. Bacteria were cultured in LB broth followed by overnight incubation at 37 °C with shaking at 180 rpm [21]. XLT4 (Difco Laboratories, San Jose, CA, USA) and 1.5% LB agar were used for bacterial culture and counts of colony-forming units (CFU).

Human epithelial cells Caco-2 BBE and mouse macrophage RAW264.7 cells were cultured and maintained in Dulbecco’s modified Eagle’s medium (DMEM) containing 10% fetal bovine serum (FBS), 50 μg/ml streptomycin, and 50 U/ml penicillin.

### Experimental animals

Female BALB/c mice (8 weeks old) used for vaccination experiments were obtained from the Comparative Medical Center of Yangzhou University (Yangzhou, China). The animal experiments were all approved by the Animal Care and Ethics Committee of Yangzhou University, Yangzhou, China (Approval ID: SYXK [Su] 2012–0029).

### Assessment of bacterial virulence

The virulence of mutant C50336Δ*sptP* and wild-type strain C50336 was evaluated in BALB/c mice by oral inoculation of the mice with various doses of the bacterial strains. The morbidity and mortality of the mice were observed as previously described [22]. Briefly, water and food were withdrawn 4 h before oral gavage with 100 μl of 5% sodium bicarbonate to neutralize stomach acid; 1 h later, mice were administered 10-fold dilutions of C50336Δ*sptP* or C50336 [1 × 10^8^–1 × 10^4^ CFU in 100 μl of phosphate-buffered saline (PBS)] by oral gavage. Control mice received 100 μl of PBS via the same route. All deaths of mice were recorded over the 16-day experimental period. The LD_50_ was calculated using the Karber and Behrens method [23].

### Bacterial colonization in cells

Human epithelial cells Caco-2 BBE and mouse macrophage RAW264.7 were grown in DMEM with 10% FBS. At 90–100% confluence, the monolayers were washed three times with PBS, and then the cells were colonized with an equal number of the indicated bacteria for 30 min (multiplicity of infection = 100). For bacterial adhesion, the cells were washed, and then incubated with PBS containing Triton X-100 (0.5%) at 37 °C for 10 min. For bacterial invasion, 30 min after bacterial colonization, the cells were incubated for an additional 30 min in DMEM with gentamicin (100 μg/ml), washed and incubated with PBS containing Triton X-100 (0.5%) at 37 °C for 10 min [24]. Serial 10-fold dilutions of cell lysates were plated on XLT4 agar and incubated at 37 °C for 12–16 h. The number of bacteria was counted and expressed as 10^2^ CFU/ml.

### Bacterial colonization and persistence in organs and tissues

Bacterial colonization and persistence in the internal organs of infected mice were evaluated. Mice were administered 1 × 10^8^ or 1 × 10^7^ CFU of C50336Δ*sptP* by oral gavage, and six mice per day were sacrificed at 1, 3, 5, 7, 14, 21, 28, and 44 days post-immunization (DPI). Liver, spleen, and mesenteric lymphadenitis (mLN) samples were aseptically collected, weighed, and homogenized in 1 ml of PBS; Serial 10-fold dilutions of tissue homogenates (100 μl each) were plated on XLT4 agar and incubated at 37 °C for 12–16 h. Bacteria were counted and the numbers are expressed as log_10_ CFU/g.

### Changes in body weight and clinical symptoms after immunization

Mice were immunized with C50336Δ*sptP* at 1 × 10^8^ or 1 × 10^7^ CFU in 100 μl of PBS by oral gavage (two doses of immunization at 0 and 14 DPI). Control animals received 100 μl of PBS. Body weight was recorded at 1, 3, 5, 7, 14, 21, 28, and 44 DPI. The mice were monitored from 1 to 44 DPI for clinical symptoms, including feed intake, susceptibility, depression and diarrhea.

### Serum IgG test

Specific IgG antibody and IgG subtype levels were measured by enzyme-linked immunosorbent assay (ELISA) using soluble antigens prepared from *S.* Enteritidis strain C50336 (SEAgP) as the coating antigen in 96-well plates (100 μl of SEAgP at 2 μg/ml in each well). Serum samples were collected from mice at 12 and 26 DPI and diluted 1:50 for use as the primary antibody. Horseradish peroxidase (HRP)-conjugated goat anti-mouse IgG (or anti-mouse IgG1 and IgG2a for IgG subtype detection) was used as the secondary antibody (dilution 1:10,000). HRP activity was determined using 3,3′,5,5′-tetramethylbenzidine (Sigma-Aldrich, St. Louis, MO, USA). Absorbance was read at 450 nm using an automated microplate reader (Titertek Multiskan; Flow Laboratories, Lugano, Switzerland).

### Lymphocyte stimulation test

The lymphocyte proliferation assay was performed using SEAgP as a stimulator. The spleen was isolated from the mice at 12 and 26 DPI and homogenized. Splenic lymphocytes were obtained by passing the homogenate through a filter with 40-μm pores (BD Biosciences, San Jose, CA, USA). Cell viability was determined based on Trypan Blue dye exclusion. Spleen mononuclear cell suspensions (1 × 10^7^ cells/ml) were cultured in Roswell Park Memorial Institute 1640 medium containing 10% FBS, 50 μg/ml streptomycin, and 50 U/ml penicillin in 96-well tissue culture plates with 10 μg/ml SEAgP or 10 μg/ml concanavalin A (Con A, a lymphocyte mitogen) as a positive control at 37 °C in a humidified atmosphere of 5% CO_2_ for 72 h. Lymphocyte proliferation was measured with a BrdU kit (Roche, Basel, Switzerland). Blastogenic responses to SEAgP are expressed as a mean stimulation index (SI) calculated based on the optical density of stimulated cultures at 450 nm, as previously described [25, 26].

### Immune protection assessment of C50336Δ*sptP*

The protective efficacy of the mutant C50336Δ*sptP* was evaluated in mice immunized orally with 1 × 10^8^ CFU (group A) in 100 μl of PBS (two doses of immunization at 0 and 14 DPI). Control mice (groups B and C, respectively) received 100 μl of PBS. At 28 DPI, group A was challenged orally with 1 × 10^8^ CFU of wild-type strain C50336 in 100 μl of PBS, whereas group B was challenged with 5 × 10^5^ CFU of wild-type strain C50336 in the same way. Group C received 100 μl of PBS as a blank control. The number of surviving mice and extent of bacterial colonization in internal organs were determined at 16 days post challenge. The number of CFUs recovered from tissues was replica-verified by PCR analysis (Primers: Forward 5′-atccgaactactttacgc-3′; Reverse 5′-tgaatggtattctactgg-3′). Clinical symptoms, including anorexia, diarrhea, depression, and mortality, were recorded daily.

### Statistical analysis

Data are expressed as the mean ± SEM. All statistical tests were two-sided. *P* < 0.05 was considered to be statistically significant. Differences between two samples were evaluated using Student’s t test. Statistical analyses were performed using SAS v.9.4 software (SAS Institute, Cary, NC, USA).

## Results

### Construction of a *sptP* deletion mutant C50336Δ*sptP* in *S.* Enteritidis

**Fig. 1:**
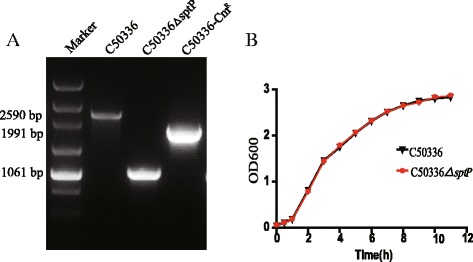
Construction of *sptP* deletion mutant in *S*. Enteritidis strain C50336. **a** PCR verification of C50336Δ*sptP* and C50336-Cm^R^. The wild type strain harbors the complete *sptP* gene, with a PCR product length of 2509 bp, whereas the PCR product from C50336Δ*sptP* has a length of 1061 bp, and the product from the C50336-Cm^R^ has a length of 1991 bp (*right*). **b** Growth curves of wild-type *S*. Enteritidis C50336 and C50336Δ*sptP*. Bacteria were grown in liquid LB medium at 37 °C for 12 h with agitation, and the OD_600_ values of triplicate cultures in LB medium were determined in 1-h intervals

The *sptP* gene deletion mutant was established in *S.* Enteritidis strain C50336 by λ-Red-mediated recombination. Our PCR data showed that the *sptP* gene was deleted in the C50336Δ*sptP* mutant (Fig. 1a). The growth characteristics of the C50336Δ*sptP* mutant and wild-type C50336 cells were determined in LB liquid medium, with no significant difference observed between the mutant and wild type (Fig. 1b). Biochemical tests were performed using the API 20E identification kit (BioMérieux, Lyon, France) and VITEK 2 Gram-negative bacilli test (BioMérieux) according to the manufacturer’s protocol, also showed no difference between these strains (data not shown).

### C50336Δ*sptP* exhibits reduced virulence in a murine model

The virulence of *S.* Enteritidis wild-type C50336 and C50336Δ*sptP* was evaluated in BALB/c mice with oral gavage. As shown in Table 1, the LD_50_ of C50336 was 3.16 × 10^5^ CFU, but no mice died in the group challenged by C50336Δ*sptP*, indicating that the virulence of the *S.* Enteritidis mutant C50336Δ*sptP* was attenuated.

**Table 1:** LD_50_ of C50336Δ*sptP* in BALB/c mice

Strain	Inoculation dose (CFU)	Number of dead mice/total number of mice	LD_50_
C50336	1.0 × 10^7^	5/5	3.16 × 10^5^
	1.0 × 10^6^	4/5	
	1.0 × 10^5^	1/5	
	1.0 × 10^4^	0/5	
C50336△*sptP*	1.0 × 10^8^	0/5	—
	1.0 × 10^7^	0/5	
	1.0 × 10^6^	0/5	
	1.0 × 10^5^	0/5	
	1.0 × 10^4^	0/5	

### *sptP* deletion leads to reduced bacterial colonization in cells

**Fig. 2:**
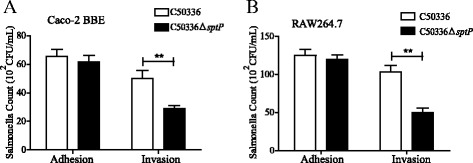
Bacterial colonization in cells. *Salmonella* adhesion and invasion in the Human epithelial Caco-2 BBE cells (**a**), and mouse macrophage RAW264.7 cells (**b**). The number of *Salmonella* was determined and presented as 10^2^ CFU/mL. Data are expressed as the mean ± SEM. **P ≤ 0.01

The bacterial colonization in cells was determined in human epithelial Caco-2 BBE and mouse macrophage RAW264.7 cells. As shown in Fig. 2, the total number of associated bacteria was not different between C50336Δ*sptP* and C50336 in Caco-2 BBE and RAW264.7 cells. However, cells colonized with C50336Δ*sptP* showed a decreased intracellular bacterial load compared to cells colonized with C50336 **(**Fig. 2a & b**)**. These results indicate that *S.* Enteritidis with *sptP* deletion has reduced bacterial colonization in vitro.

### Colonization and persistence of *Salmonella* C50336Δ*sptP* in internal organs and tissues

**Fig. 3:**
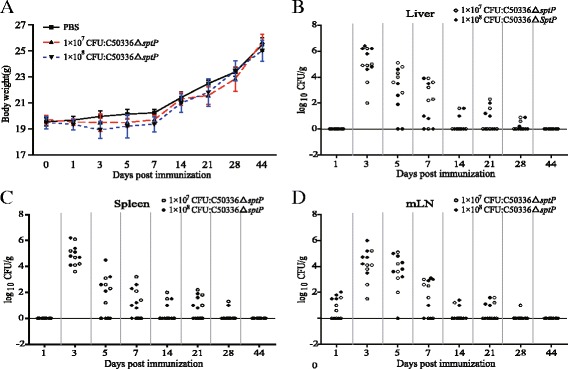
Body weight (**a**) and bacterial colonization in mouse organs post immunization. *Salmonella* colonization and persistence in the liver (**b**), spleen (**c**), and mLN (**d**) of mice following oral immunization with 1 × 10^7^ CFU or 1 × 10^8^ CFU of C50336Δ*sptP*. Control mice received 100 μl of PBS and were negative for *Salmonella* in the liver, spleen, and mLN. The number of *Salmonella* was determined and represented as Log_10_ CFU/g

The bacterial number was calculated in the liver, spleen and mLN of BALB/c mice following oral immunization with 1 × 10^8^ CFU or 1 × 10^7^ CFU C50336Δ*sptP*. As shown in Fig. 3, *Salmonella* colonization reached the highest level at 3 DPI (first immunization) in the liver, spleen, and mLN (Fig. 3b–d), and at 28 DPI (14 days post second immunization), two and one of the six mice were positive for *Salmonella* in the liver and spleen, respectively, in both the 1 × 10^8^ and 1 × 10^7^ CFU- immunized groups. In the 1 × 10^7^ CFU immunized group, *Salmonella* was detected in the mLN of one mouse. At 44 DPI, we did not detect *Salmonella* in any tissues from immunized mice. All samples from the negative control group (PBS group) were negative for *Salmonella*.

### Immunogenicity of C50336*ΔsptP*

**Fig. 4:**
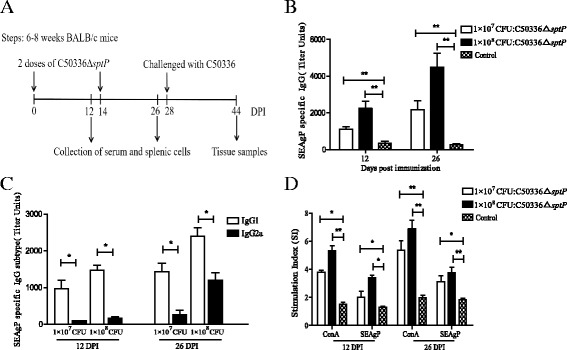
Immunogenicity of C50336Δ*sptP*. Procedure for immunization (**a**). Determination of serum IgG levels (**b**) and IgG subtype levels (**c**) at 12 and 26 days post immunization. Stimulation index (SI) of mice lymphocytes determined by splenic lymphocyte proliferation assay using soluble antigen SEAgP (**d**) or lymphocyte mitogen ConA as a positive control. The vaccinated group received 1 × 10^7^ CFU or 1 × 10^8^ CFU of C50336Δ*sptP* orally, and the control group received 100 μl of PBS. Data are expressed as the mean ± SEM. *P ≤ 0.05, **P ≤ 0.01

The changes in body weight and clinical symptoms were monitored in mice following immunization with C50336Δ*sptP*. The body weights of the mice were shown in Fig. 3a. A decrease in body weight loss was noticed in the initial days of post immunization, but no statistically significant differences were observed among the three groups (immunized with 1 × 10^8^ CFU C50336Δ*sptP*, immunized with 1 × 10^7^ CFU C50336Δ*sptP*, and treated with PBS as a control). No significant differences were found in clinical symptoms among the three groups, although slight and temporary anorexia was observed in vaccinated mice. Clinical signs of disease such as lethargy and diarrhea were absent in all immunized mice and control mice. The procedures for immunization are shown in Fig. 4a.

The humoral immune responses were evaluated in mice following oral immunization with C50336Δ*sptP*. Serum IgG levels were determined by indirect ELISA. As shown in Fig. 4b, mice immunized with 1 × 10^8^ CFU and 1 × 10^7^ CFU of C50336Δ*sptP* showed significantly higher levels of serum IgG at 12 and 26 DPI than the control group. Furthermore, the titers of the IgG1 subtype were significantly higher than those of IgG2a, demonstrating that *S*. Entertidis C50336Δ*sptP* tends to induce a Th2 immune response in mice (Fig. 4c).

The cellular immune responses in mice following oral immunization with C50336Δ*sptP* were evaluated by splenic lymphocyte proliferation assay. As shown in Fig. 4d, the SI values of immunized mice were significantly higher than control mice after stimulation with SEAgP or lymphocyte mitogen ConA at 12 and 26 DPI. This finding indicates that C50336Δ*sptP* also induces cellular immune responses in mice.

### C50336Δ*sptP* protects mice against oral challenge with wild-type *S*. Enteritidis

**Fig. 5:**
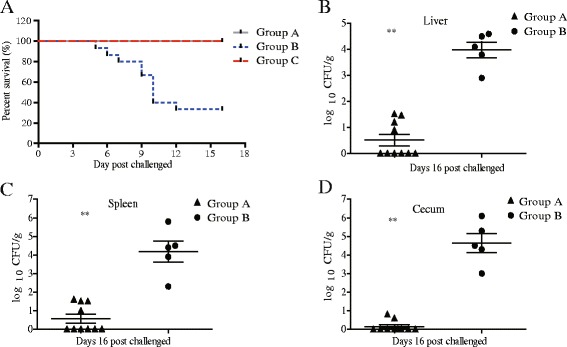
Survival curve (**a**) and bacterial colonization in challenged mice. Bacterial colonization in the liver (**b**), spleen (**c**) and cecum (**d**) of challenged mice (Table 2, group A, immunized with C50336Δ*sptP* and then challenged with 1 × 10^8^ CFU of C50336; group B, immunized with PBS and then challenged with 5 × 10^5^ CFU of C50336). All samples from the blank control group were negative for *Salmonella* (Table 2, group C). The number of bacteria was determined and expressed as log_10_ CFU/g. Data are expressed as the mean ± SEM, **P ≤ 0.01

Mice were orally vaccinated with 2 doses of 1 × 10^8^ CFU of C50336Δ*sptP*, and then at 28 DPI, they were challenged with wild-type C50336. The percentage of surviving mice at 16 days post challenge is shown in Table 2. None of the immunized mice died in group A and group C, whereas ten of fifteen mice died in control group B. Slight and temporary anorexia as well as depression were observed following challenge in the immunized mice (group A) when compared the blank control group C. Severe clinical symptoms and mortality were observed following challenge in the non-immunized mice. The survival curve is shown in Fig. 5a.

**Table 2:** Protective effects of C50336Δ*sptP* in mice

Group	Vaccination		Number	Challenge			Survivors/total	Survival rate (%)
	Strain	Dose (CFU)		Strain	Route	Dose (CFU)		
A	C50336Δ*sptP*	1 × 10^8^	10	C50336	oral	1 × 10^8^	10/10	100
B	PBS	—	10	C50336	oral	5 × 10^5^	5/15	33
C	PBS	—	10	PBS	oral	—	5/5	100

All surviving mice were sacrificed at 16 days post challenge, and tissues including the liver, spleen and cecum were sampled. As shown in Fig. 5, for the group vaccinated with 1 × 10^8^ CFU of C50336Δ*sptP*, four of ten immunized mice carried a low level of *Salmonella* in the liver and spleen (Fig. 5b & c), and two of ten mice were positive for *Salmonella* in the cecum (Fig. 5d). The mice in group B carried a higher level of *Salmonella* in the liver, spleen, and cecum than the immunized mice from group A. All samples from the blank control group (group C) were negative for *Salmonella*.

## Discussion

In current study, we constructed an *sptP* mutant in *S.* Enteritidis C50336, and evaluated the efficacy of C50336Δ*sptP* as a candidate live attenuated vaccine for *Salmonella* infection based on virulence, changes in body weight and clinical symptoms, bacterial colonization, serum IgG level, splenic lymphocyte proliferation and protective efficiency in mice. Our results showed that the body weight change of mice orally vaccinated with C50336Δ*sptP* (1 × 10^7^ CFU or1 × 10^8^ CFU) was similar to that of the PBS control. After immunization with C50336Δ*sptP*, only small amounts of *Salmonella* colonized and persisted in the liver and spleen of the mice at 28 days, but strong humoral and cellular immune responses were induced to protect the mice against secondary *S*. Enteritidis challenge.

*Salmonella enterica* subsp. enterica includes several important serovars including Typhimurium, Enteritidis Typhi, and Paratyphi, which are the leading sources of human salmonellosis. Vaccines have been developed for one of these serovars and will prevent disease caused by *S*. Typhimurium or *S*. Enteritidis [27]. Symptoms of human Salmonellosis include abdominal pain, diarrhea, nausea, vomiting, fever, and headache [5]. *S*. Enteritidis is also increasingly reported in cases of invasive and extra-intestinal infections, such as arthritis, septicemia, meningitis, endocarditis, and urinary tract infections [28–34]. Infection with *Salmonella* is usually caused by consumption of contaminated pork and beef, poultry and eggs. It is necessary to take effective measures to control and prevent *Salmonella* infection. Vaccination with live attenuated *Salmonella* may be a viable choice. Many live attenuated vaccines have been developed against *Salmonella* and have been confirmed to be generally more effective than vaccines prepared with dead bacteria [9–14].

The ideal vaccine should be avirulent, especially for live attenuated vaccines. Some *S*. Typhimurium and *S*. Enteritidis mutants with deletion of T3SS effector encoding genes or SPI-1 or SPI-2 display decreased virulence in mice, poultry, pigs, and humans [35–40]. In the current study, the LD_50_ of C50336Δ*sptP* in mice inoculated by the oral route demonstrated that the virulence of the mutant C50336Δ*sptP* was significantly decreased in comparison with that of the wild-type strain. The body weight of mice immunized with C50336Δ*sptP* was not significantly different from that of control mice and no or less clinical symptoms were observed following immunization. All of these findings show that C50336Δ*sptP* has almost no side effects in terms of growth performance in mice.

For live attenuated *Salmonella* vaccines, it is critical to stimulate the humoral and cellular immune responses in the immunized host [8, 41]. Systemic dissemination of *Salmonella* can lead to antigen presentation, resulting in high induction of specific humoral and cellular immune responses, and efficient protection against secondary infection [12, 42]. In this study, the *Salmonella*-specific serum IgG level in mice immunized with C50336Δ*sptP* at 12 and 26 DPI was significantly higher than the antibody level detected in the control group. In addition, the SI values of splenic lymphocyte proliferation in immunized mice were significantly higher than that in control mice. All of these findings indicate that strong humoral and cellular immune responses can be stimulated by C50336Δ*sptP*. In addition, the mice vaccinated with 1 × 10^8^ CFU C50336Δ*sptP* showed higher levels of serum IgG and splenic lymphocyte proliferation capability than mice vaccinated with 1 × 10^7^ CFU C50336Δ*sptP*.

*S*. Enteritidis deleted with one or several effector encoding genes provided efficacious protection against secondary *S*. Enteritidis challenge in mice [12, 13]. In our study, to evaluate the protective efficacy of C50336Δ*sptP*, we orally immunized mice with 1 × 10^8^ CFU of C50336Δ*sptP*, and determined protection rates following oral challenge with 1 × 10^8^ CFU of wild-type C50336. Our results show that mice immunized with 1 × 10^8^ CFU of C50336Δ*sptP* developed extensive humoral and cellular immune responses and experienced 100% protection against subsequent *S*. Enteritidis challenge.

Pathogenesis of *Salmonella* is associated with a specialized ability to invade and persist within intestinal epithelial cells, where it can replicate and evade the host immune system [43]. T3SS-1 and T3SS-2 are encoded by SPI-1 and SPI-2 respectively, secreting various effector proteins that are essential for *Salmonella* colonization and intracellular persistence in the host [11, 35, 44]. SptP is a T3SS-1 effector protein that mediates the reversion of pathogen-induced changes in the actin cytoskeleton after *Salmonella* invasion [15]. In our study, *S.* Enteritidis C50336 with deletion of *sptP* led to decreased invasion in Caco-2 BBE and RAW264.7 cells. SptP also inhibits the MAPK pathway, suppresses IL-8 production, and reduces the inflammatory response in host cells, thereby enhancing intracellular *Salmonella* replication [16–18]. In current study, deletion of *sptP* resulted in induction of strong humoral and cellular immune responses against subsequent *Salmonella* challenge.

## Conclusion

Our present work demonstrates that vaccination of mice with the candidate vaccine C50336Δ*sptP* conferred development of acquired immunity and efficacious protection against experimental systemic infection. Thus, the *sptP* mutant strain of *S*. Enteritidis C50336 has the potential of being a safe, immunogenic vaccine against *Salmonella* infection.

## Abbreviations

CFU: Colony-forming units
DPI: Days post-immunization
ELISA: Enzyme-linked immunosorbent assay
IL-8: Interleukin-8
LD_50_: 50% lethal dose
mLN: mesenteric lymphadenitis
PBS: phosphate-buffered saline
*S*. Enteritidis: *Salmonella enterica* serovar Enteritidis
SI: Stimulation index
SPI-1: *Salmonella* pathogenicity islands 1
SptP: *Salmonella* protein tyrosine phosphatase
T3SS: *Salmonella* Type III secretion system
